# Prediction of evolutionarily conserved interologs in *Mus musculus*

**DOI:** 10.1186/1471-2164-9-465

**Published:** 2008-10-08

**Authors:** Sailu Yellaboina, Dawood B Dudekula, Minoru SH Ko

**Affiliations:** 1 Developmental Genomics and Aging Section, Laboratory of Genetics, National Institute on Aging, National Institutes of Health, Baltimore, MD 21224, USA

## Abstract

**Background:**

Identification of protein-protein interactions is an important first step to understand living systems. High-throughput experimental approaches have accumulated large amount of information on protein-protein interactions in human and other model organisms. Such interaction information has been successfully transferred to other species, in which the experimental data are limited. However, the annotation transfer method could yield false positive interologs due to the lack of conservation of interactions when applied to phylogenetically distant organisms.

**Results:**

To address this issue, we used phylogenetic profile method to filter false positives in interologs based on the notion that evolutionary conserved interactions show similar patterns of occurrence along the genomes. The approach was applied to *Mus musculus*, in which the experimentally identified interactions are limited. We first inferred the protein-protein interactions in *Mus musculus *by using two approaches: i) identifying mouse orthologs of interacting proteins (interologs) based on the experimental protein-protein interaction data from other organisms; and ii) analyzing frequency of mouse ortholog co-occurrence in predicted operons of bacteria. We then filtered possible false-positives in the predicted interactions using the phylogenetic profiles. We found that this filtering method significantly increased the frequency of interacting protein-pairs coexpressed in the same cells/tissues in gene expression omnibus (GEO) database as well as the frequency of interacting protein-pairs shared the similar Gene Ontology (GO) terms for biological processes and cellular localizations. The data supports the notion that phylogenetic profile helps to reduce the number of false positives in interologs.

**Conclusion:**

We have developed protein-protein interaction database in mouse, which contains 41109 interologs. We have also developed a web interface to facilitate the use of database .

## 1 Background

Many functions in living organisms are determined by interactions among proteins in cells. Identifying these interactions is an important first step in systems level understanding of various developmental, physiological, and disease processes. High-throughput experimental approaches such as yeast two-hybrid system and tandem affinity purification coupled with mass spectrometry have been carried out to map protein-protein interactions in model organisms [1-6]. These experimental data have been curated to produce protein-protein interaction databases such as BIOGRID [7], INTACT [8], MINT [9], DIP [10], and Reactome [11]. Computational methods have also been developed to transfer the interaction annotation from one organism to another through identifying orthologs by comparative genomics methodology [12,13].

In addition to the experimental approaches mentioned above, a number of algorithms have been developed to predict protein-protein interactions by computationally analyzing completely sequenced genomes. Some of these algorithms identify the interactions between proteins on the basis of chromosomal proximity of two genes. These methods rely on the notion that genes encoding functionally interacting proteins show conserved gene neighborhood and are often localized in gene clusters or operons in the bacterial genomes [14-17]. Special case of chromosomal proximity is a gene-fusion, where the fusion between two genes in another genome is usually a strong indication for a physical interaction between the proteins encoded therein [18]. Regardless of the proximity in the chromosome, being encoded in the same genome and their co-evolution can be a prerequisite for functional interaction. One such approach is a phylogenetic profile method that identifies interactions by using the pattern of occurrence of genes or protein domains in genomes of different species [19-21]. Other coevolution methods are an *in-silico *two hybrid system and mirror tree method, which detects interactions between the proteins on the basis of correlated mutations and similarity of phylogenetic trees, respectively [22].

A potential problem in predicting protein-protein interactions using such an interolog-based method is that it may generate false positive interactions, because of false positives in the original high-throughput interaction data [23,16] and false positive interologs due to the lack of evolutionary conservation of interactions when applied to phylogenetically distant organisms [24]. Topology of the network (quasi clique score) has been used to filter out the false positives interologs on the basis of notion that the highly interconnected proteins are likely to be evolutionary conserved [25]. For accurate transfer of interactions to orthologs, HomoMINT uses domain matching algorithm to filter the false positives in orthologs [26]. Because the interacting proteins are likely to show similar functions, functional similarity of gene ontology terms has been used to reduce the false positives in high-throughput protein-protein interaction data [27,28].

In the present work, we inferred the interactions between *Mus musculus *proteins, if their orthologs are known to be interacting in other species or part of predicted operons in bacteria. We anticipated the presence of false positives in predicted interactions due to the lack of evolutionary conservation of interactions. To reduce the false positives, we have used the phylogenetic profiles of interacting proteins and filtered out unlikely interactions.

## 2 Results and discussion

**Figure 1:**
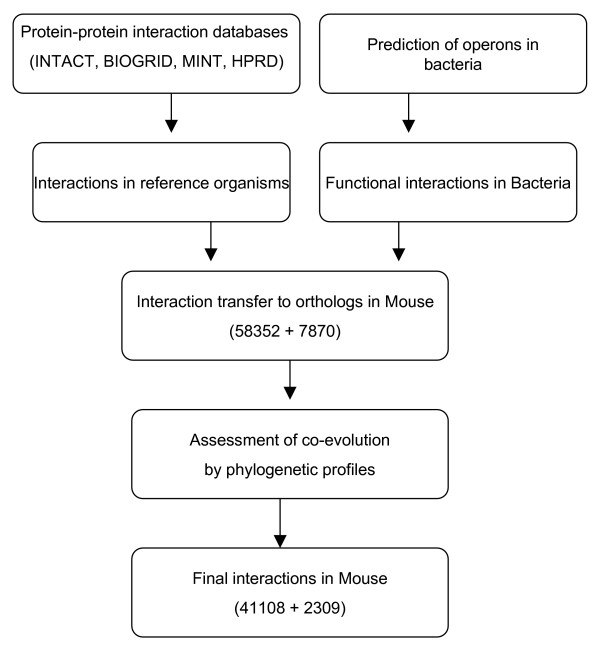
**Flowchart of the approach used to predict protein interactions in Mus musculus.** Protein interactions were generated using two approaches; 1) Physical interactions in different databases 2) functional interactions in operons. The interactions were transferred to orthologs of Mus musculus and false positives in the interactions were filtered using phylogenetic profiles.

Figure 1 shows a flowchart for over all approaches.

### 2.1 Transferring experiment-based interologs of model organisms to *Mus musculus*

We downloaded all experimentally-identified protein-protein interactions from BIOGRID [7], INTACT [8], MINT [9], and HPRD [29]. The majority of the reported interactions in these databases come from *Homo sapiens *and experimental model organisms such as *Rattus norvegicus, Drosophila melanogaster, Saccharomyces cerevisiae, Caenorhabditis elegans, Arabidopsis thaliana*, and *Escherichia coli K12 *(called reference organisms) (Table 1). The gene symbols and aliases for each reference organism were obtained from the NCBI Gene database . Based on the NCBI gene annotation, the interactions from the different databases were transferred to the reference organisms. We then prepared a set of non-redundant interactions for each reference organism by merging the different PubMed IDs for the same interaction.

**Table 1:** Distribution of interologs in mouse before and after filtering with phylogenetic profiles

	Non-redundant interactions identified experimentally in each species	Interologs transferred to mouse based on orthologous relationship	Interologs remained after filtering by phylogenetic profiles	Fraction of filtered interologs (%)
*Escherichia coli K12*	*13734*	1261	294	76%

*Saccharomyces cerevisiae*	*62965*	19605	12528	36%

*Caenorhabditis elegans*	*4663*	924	627	32%

*Arabidopsis thaliana*	*915*	21	20	5%

*Drosophila melanogaster*	*10788*	2506	1876	25%

*Rattus norvegicus*	*881*	790	373	52%

*Mus musculus*	*2979*	2979	2224	25%

*Homo sapiens*	*48276*	30806	23166	25%

We transferred the interactions from each of the reference organisms to *Mus musculus *on the basis of orthology relationship predicted as the best hit by bi-directional BLASTp [30] searches against all proteins using an 10^-4 ^as a cut-off e-value. As for the redundant interologs in mouse, an interolog from evolutionarily more closely related species was selected. Table 1 shows the combined non-redundant set of original interactions for each reference organism and *Mus musculus*. *Homo sapiens *contributed the greatest number of interologs to *Mus musculus*, followed by *Saccharomyces cerevisiae*. After removing redundancy, we obtained a total 55913 non-redundant interologs in *Mus musculus*. The final number of interactions consisting of interologs and interactions identified experimentally in *Mus musculus *was 58352 [See Additional file 1].

### 2.2 Predicting interactions based on the co-occurrence of mouse orthologs in predicted bacterial operons

We used the support vector machine (SVM) that was trained on intergenic distances to predict the operons in 186 species of bacteria, as described previously [16] [See Additional file 2]. When two mouse orthologs were found at least in one predicted operon, we considered these two mouse proteins interacting either functionally or physically. Using this method, we identified 7870 interactions between 2054 proteins in *Mus musculus *[See Additional File 3]. In general, the reliability of the interaction decreases as ortholog frequency of co-occurrence in predicted operons decreases. To make this point clear, we sorted the interactions by the decrease in frequency of co-occurrence in predicted operons and showed it in the column 5 in Additional file 3.

**Figure 2:**
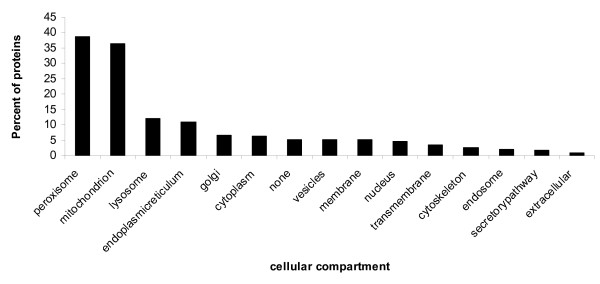
**Percent distribution of organelle proteins in the interactions dataset predicted by ortholog co-occurrence in operons.** Localization information is obtained from eSLDB (Pierleoni, et al., 2007) . Categories of sub cellular localization are defined according to the Swiss-prot annotation. Protein sequences with no localization information are named as 'None'.

By analyzing the cellular location of these interacting proteins by Gene Ontology (GO) terms, we found that most of the peroxisomal and mitochondrial proteins were included in this set of interacting proteins (Figure 2). This seems to support the notion that the prokaryotes are ancestors of mitochondria [31]. The predicted interactions will thus be useful to understand their biological and disease processes in peroxisome and mitochondria.

We combined 58352 experiment-based interologs and 7870 operon-based interactions, removed redundancy, and obtained 65515 protein-protein interactions in *Mus musculus*. Relatively low overlap (707 common interactions) between experiment-based interologs and operon-based interologs may possibly be due to the scarcity of the known interactions.

### 2.3 Filtering false positives using phylogenetic profiles

The interactions transferred from both experimentally identified interactions from model organisms (section 2.1) and predicted operons of bacteria (section 2.2.) may include false positives due to false positives in original interactions obtained experimentally or the lack of evolutionary conservation of interactions. We, therefore, used a phylogenetic profile of 26 eukaryotic and 186 bacterial species to filter possible false positives in the predicted interactions. The reason for including bacterial species for the analysis was that many interactions were derived from predicted operons of bacteria. Furthermore, some orthologous proteins in experimentally identified interactions for eukaryote model organisms can be found in bacterial species.

We first built a model by training the SVM on phylogenetic profiles of positive and negative protein-protein interaction datasets. The positive data for protein-protein interactions were taken on the basis of evolutionary conservation of interactions and the number of experimental observations of interactions using the PubMed IDs. Conserved interactions or interologs that are present in more than one species are likely to be true interactions or interologs. The interactions observed by multiple experiments are also likely to be true interactions. There were 1637 interologs present in multiple species and 6043 interologs in mammals (*Mus musculus, Rattus norvegicus*, and *Homo sapiens*) with multiple PubMed IDs. After merging both datasets and removing the redundancy, the final dataset contained 7308 protein pairs corresponding to the 6348 gene pairs. The negative dataset for predicting functional linkages was assumed to be those proteins that are not co-localized in the same sub-cellular compartment [32]. The protein localization data for *Mus musculus *was obtained from eSLDB [33]. The negative dataset of protein interactions was prepared by the pairwise combination of proteins from nucleus/mitochondria and extracellular space. The negative dataset contained 708060 protein pairs corresponding to 657924 gene pairs.

**Figure 3:**
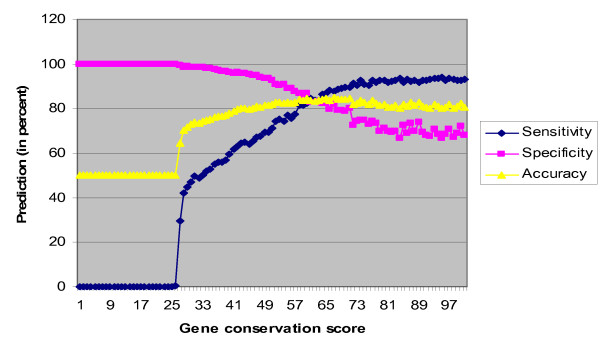
**Effect of gene conservation score on accuracy of predictions using phylogenetic profiles.** Accuracy is defined as the average of sensitivity and specificity as described in Methods. It is clearly seen that the prediction accuracy is poorer at low conservation scores and maximum at the conservation score of 58. See text for details.

Bit scores for homologs of all the proteins of *Mus musculus *were obtained by protein BLAST search [30] against proteomes of 213 species. The phylogenetic profile of a gene is represented as a normalized bit score profile of its encoded protein [34]. The protein phylogenetic profile was converted to a gene phylogenetic profile, because there are no representative symbols for most of the protein sequences in the NCBI database and the most of the protein names in interaction databases are represented by gene symbols. If a gene encodes multiple proteins by alternative splicing, the profile of a protein with the greatest conservation score was selected. We believe that this treatment is reasonable, because the predictive power of phylogenetic profile method increases with the increase of conservation score of a protein [16]. Similarity of the phylogenetic profiles was assessed on the basis of Pearson correlation coefficient. One problem we encountered was that Pearson correlation coefficient tended to show high scores, if two genes that we compared were not evolutionarily well conserved, but present in some specific lineages. Therefore, negative dataset in the training set sometimes produced high scores, resulting in the false negatives during prediction. To examine this further, we generated different negative datasets by varying conservation scores and assessed the effect of negative training data on the accuracy of prediction (See methods section for the definition of accuracy used here) (Figure. 3).

The conservation score for a gene is defined as a total number of genomes in which gene homologs are found [35]. The conservation score of a pair of genes was in turn defined as the least conservation score of any of two genes in pairs. To understand the effect of conservation score of a gene pair on prediction accuracy in the phylogenetic profile method, we used a half of randomly picked data from the positive and negative data sets for training SVM and the remaining half for testing the prediction accuracy. The process was repeated 100 times with each time by incrementing the cut-off conservation score by 1 in the negative data set while retaining the same positive data set. Figure 3 shows that the prediction accuracy of protein-protein interactions reached the maximum at the conservation score of 59.

We observed a similar trend when we plotted a true positive rate (i.e., "sensitivity") versus a false positive rate (i.e., "1 – specificity") in a receiver operator characteristic (ROC) graph [See Additional file 4]. As expected, we found that the accuracy value became maximum, when the "sensitivity" (0.82 on y-axis) and "1 – specificity" (0.13 on x-axis) values were at the point near the upper left corner. The accuracy remained constant beyond the conservation score of 59, and therefore, we considered phylogenetic profiles of genes in the negative dataset with the conservation score greater than 59. The number of negative dataset with a conservation score greater than 59 was 4454.

The best model for the prediction of protein-protein interactions was selected using the standard five-fold cross validation. Positive and negative datasets were randomly divided into five groups. One-fifth was used as a test set and remaining four-fifth was used as a training set. This was repeated five times with a different set of one-fifth used for testing each time. Additional file 5 shows the specificity, sensitivity, and accuracy at each trial of cross validation. Prediction accuracy at each trial of the cross validation was consistent with each other, indicating the homogeneity of the training dataset. The average accuracy was 84.1%. A model generated with the highest accuracy was retained as the best model, which was used to predict the positives and false positives in the interactions predicted above.

Among 7870 predicted interactions by ortholog co-occurrence in operons, 2309 were predicted as true interactions on the basis of phylogenetic profiles [See Additional file 6]. Similarly, among 58352 interactions, 41109 were predicted as true interactions using phylogenetic profiles [See Additional file 7]. In Table 1, Column 2 shows the final number of interologs in mouse from each reference organism, whereas Column 3 shows the fraction of interologs that were filtered out from each reference organism by phylogenetic profiles. The fraction of interologs that were predicted as false positives using phylogenetic profiles gradually increased with evolutionary distance between mouse and a reference organism. Exceptions were *Arabidopsis thaliana *and *Rattus norvegicus*: this might be caused by the fewer number of available interactions in the datasets.

### 2.4 Evaluation of predicted interactions

To validate the final protein-protein interaction datasets, we used the notion that interacting proteins should share the same subcellular localization, have often similar functions, and are co-expressed in the same tissues. Subcellular co-localization and functional similarity of interacting proteins were assessed by the similarity in Cellular Compartment (CC) and Biological Process (BP) GO terms, respectively. The function "getGeneSim" in GOSim package was used for similarity measure [36]. Frequency of co-expression of *Mus musculus *genes in GEO microarray data were calculated as described in the methods section. The distributions of co-expression frequency and gene ontology (CC, BP) similarity scores were significantly different between interologs filtered with phylogenetic profiles and false positive interologs (Wilcoxon test P value < 2.2e-16). As shown in Table 2, the interologs filtered with the phylogenetic profile showed the highest mean value of co-expression frequency and similarity between gene ontology terms (CC and BP), when compared to the interologs that were not filtered and the negative data set. Furthermore, mean value of co-expression frequency and similarity between gene ontology terms of interactions predicted by mouse ortholog co-occurrence in bacterial operons showed the highest mean value when compared to the interologs and the negative data set. This suggests that the protein-protein interactions obtained after filtering with the phylogenetic profiles are more reliable than those obtained without filtering.

**Table 2:** Evaluation of predicted interactions by frequency of co-expression and functional similarity of GO terms

	Frequency of co-expression (Mean/Stddev)	Similarity of GO term (BP) Mean/Stddev)	Similarity of GO term(CC)(Mean/Stddev)
**Protein – protein interaction datasets**			

Interolog	3.7/5.9	0.32/0.21	0.40/0.31

interolog + phylogenetic profile	4.18/6.45	0.34/0.22	0.43/0.31

Interologs predicted as false positives by phylogenetic profiles	2.70/4.58	0.29/0.17	0.34/0.30

Ortholog co-occurrence in operons	5.0/8.8	0.41/0.25	0.28/0.35

Ortholog co-occurrence in operons + phylogenetic profile	10.11/15.76	0.51/0.27	0.46/0.37

**Negative data**			

	0.48/1.74	0.23/0.14	0.37/0.24

The interaction datasets were evaluated by co-expression frequency of interacting genes and similarity between gene ontology terms BP (Biological Process) and CC (Cellular component).

See Methods section for the details of co-expression frequency. Similarity between GO terms was calculated by using "getGeneSim" function in GOSim package [36].

### 2.5 Web interface for data browsing

To provide a user-friendly access to the database, we developed a WWW interface that allows one to search for the potential protein interactions for a gene or a list of gene . Users can select a type of protein interaction dataset and enter names of genes as an Entrez gene ID, gene symbol, GenBank accession number for nucleotide and proteins, or NIA Mouse Gene Index U cluster IDs. Genes have also been directly linked to the NIA Mouse Gene Index [37]. The web interface returns results as a network diagram [38] and a table that lists information on individual interactions, such as a method of identification, protein domains, species conservation, co-occurrence of gene symbols in PubMed abstracts, and protein localization. All the data are also available for download at our website .

## 3 Conclusion

Interactions between proteins in *Mus musculus *were inferred on the basis of their orthologous interaction information in other organisms and the functional linkage information in predicted operons of bacteria. Possible false-positives in these interactions were filtered out using phylogenetic profiles on the basis of the notion that the evolutionarily conserved interactions should show similar pattern of occurrence along the genomes. Information about protein-protein interactions with high confidence will be useful to understand various processes in mammalian model organism, *Mus musculus*. Predicted interactions based on bacterial operons will provide useful insights into the function of mammalian mitochondrial proteins and their functional interactions. A web interface provides access to the database for a variety of investigations, including DNA microarrays and proteomics researches.

## 4 Methods

The proteomes and completely sequenced genome of bacteria and eukaryotes were downloaded from NCBI ftp site . The homologous sequences of all the known open reading frames (ORFs) of *Mus musculus *were searched using BLASTp [30] against the proteome of other species with 10^-4 ^as the cut off value. Orthologs of the *Mus musculus *genes were identified as the best hit by bi-directional BLASTp [30] searches against all proteins with 10^-4 ^as the cut off value. It is known that, if multiple proteomes for each species are included, phylogenetic profile produces less accurate results [39,40]. Therefore, when more than two proteome sequences for the same species were available, we selected the one that shared the maximum number of orthologs with *Mus musculus*. Finally there were 186 genomes of bacterial species and 26 genomes of eukaryotes species [See Additional file 2].

The SVM was trained on datasets for both positive and negative interactions. Pearson correlation co-efficient between the phylogenetic profiles of gene pairs was used as inputs to the SVM classifier. To validate the datasets for model selection and prediction accuracy, we have used five fold cross validation, in which the positives and negative datasets were randomly divided into five equal size sets. Training and testing carried out using the "svm-train" and "svm-predict" tools of the LibSvm software [41]. In each step of cross validation four sets are used for training and remaining one for testing. In each step of testing, sensitivity, specificity and "balanced accuracy [42]" were calculated in the following manner:

Sensitivity = (True Positives)/[(True Positives) + (False Negatives)]

Specificity = (True Negatives)/[(True Negatives) + (False Positives)]

Balanced accuracy = (Specificity + Sensitivity)/2

We used "balanced accuracy" instead of the standard overall "accuracy," because it has been reported that the accuracy becomes particularly problematic as a measure of validity, when the difference between sensitivity and specificity increases [43]. We indeed observed this problem, when we applied the standard overall "accuracy" to the data shown in Figure 3. When the sensitivity was low and the specificity was high (1 < = gene conversion score < = 26), the overall accuracy was unreasonably high (99%) (Figure 3). In contrast, the "balanced accuracy" provided more reasonable estimates even in these cases (Figure 3; See Additional file 5).

Radial basis function (RBF) was used as a kernel of the Support vector Machine (SVM). To choose kernel parameters of SVM, we carried out "grid-search" using "grid.py" of LibSVM. In "grid-search", pairs of cost (c) and gamma (γ) were tested in each step of cross validation and one with the best cross validation accuracy was picked.

The co-expression frequency of gene pairs was calculated using a method similar to the one described previously[44]. *Mus musculus *microarray datasets were downloaded from the NCBI GEO database . The datasets with a sample number less than 11 were excluded from the analysis. Finally, there were 286 datasets. Between each possible gene pair in each dataset, Pearson correlation coefficient and its p-value was calculated using the "Pearson" function described in the Numerical recipes in C [45]. A functional link between a gene pair was inferred if the Bonferroni corrected p value is less than 0.05.

## 5 Abbreviations

BP: Biological Process; CC: Cellular Compartment; FN: false negatives; FP: false positives; GEO: gene expression omnibus; GO: Gene Ontology; NCBI: National Center for Biotechnology Information; NIA: National Institute on Aging; ORF: open reading frames; RBF: Radial basis function; ROC: receiver operator characteristic; SVM: support vector machine; TP: true positives; TN: true negatives.

## Authors' contributions

SY designed the study, carried out the computation and data analysis, and drafted the manuscript; DBD designed and constructed the website; MSHK designed and coordinate the study, and completed the manuscript. All authors read and approved the final manuscript.

## Supplementary Material

Additional file 1 A list of interactions transferred from other organisms to *Mus musculus *and interactions identified experimentally in *Mus musculus*. Columns A and B show a pair of Entrez Gene IDs of interacting proteins in *Mus musculus; *Columns C and D show the corresponding GIs of interacting proteins in *Mus musculus; *Columns E and F show the corresponding GIs of interacting proteins in reference organism; Column G shows an experimental method used to identify the interaction in reference organism; Column H shows PubMed IDs of the article in which the interactions were reported; Column I shows the name of the reference organism in which the interaction was identified; and Column J shows the source of the interaction of reference organism. Click here for file

Additional file 2 A list of genomes used for phylogenetic profile analysis and operon prediction. For the phylogenetic profile analysis 26 genomes of eukaryote species and 186 genomes of bacterial species were used. The 186 genomes of bacterial species were also used for operon prediction. Click here for file

Additional file 3 A list of interactions in *Mus musculus*, which were transferred from operons of bacterial species. Columns A and B show a pair of the Entrez Gene IDs of interacting proteins in *Mus musculus; *Columns C and D show the corresponding GIs of interacting proteins in *Mus musculus; *and Column E shows the co-occurrence frequency of Mus musculus protein orthologs in bacterial operons. Click here for file

Additional file 4 ROC Graph for prediction of protein – protein interactions. The ROC curve is a plot of "sensitivity (True positive rate)" versus "1 – specificity (False positive rate)." The "sensitivity" and "1 – specificity" values at the point near the upper left corner are 0.82 and 0.13, respectively, where the balanced accuracy for prediction of protein – protein interaction reaches the maximum (86%). Click here for file

Additional file 5 Accuracy of cross validation in phylogenetic profiles. A table shows the number of true positives (TP), false negatives (FN), true negatives (TN), and false positives (FP) in each step of five-fold cross validation trials. Sensitivity and Specificity are calculated according to the formula described in the Methods section. The accuracy reaches the maximum in the 4^th ^step of cross validation trials. Click here for file

Additional file 6 A list of filtered interactions in *Mus musculus*, which were transferred from operons of bacterial species and filtered with the phylogenetic profiles method. Columns A and B show a pair of the Entrez Gene IDs of interacting proteins in *Mus musculus; *Columns C and D show the corresponding GIs of interacting proteins in *Mus musculus; *Column E shows the co-occurrence frequency of *Mus musculus *protein orthologs in bacterial operons; and Column F shows the Pearson correlation co-efficient between the phylogenetic profiles of mouse proteins. Click here for file

Additional file 7 Interologs and experimentally identified interactions in *Mus musculus *obtained after filtering with the phylogenetic profiles method. Columns A and B show a pair of the Entrez Gene IDs of interacting proteins in *Mus musculus; *Columns C and D show the corresponding GIs of interacting proteins in *Mus musculus; *Columns E and F show the corresponding GIs of interacting proteins in reference organism; Column G shows the experimental method used to identify the interaction in reference organism; Column H shows the PubMed IDs of the article, in which the interactions were reported; Column I shows the name of the reference organism, in which the interaction was identified; Column J shows the source of the interaction of reference organism; and Column K shows the Pearson correlation co-efficient between the phylogenetic profiles of mouse proteins. Click here for file
