# Supplementary material for: Prediction of evolutionarily conserved interologs in Mus musculus
Source: BMC Genomics. 2008 Oct 8;9:465. doi: 10.1186/1471-2164-9-465 (PMC2571111; doi:10.1186/1471-2164-9-465)
Supplement: Additional file 2 — A list of genomes used for phylogenetic profile analysis and operon prediction. For the phylogenetic profile analysis 26 genomes of eukaryote species and 186 genomes of bacterial species were used. The 186 genomes of bacterial species were also used for operon prediction. [file 1471-2164-9-465-S2.doc]

Eukaryotes

Anopheles_gambiae

Apis_mellifera

Arabidopsis_thaliana

Aspergillus_fumigatus

Bos_taurus

Caenorhabditis_elegans

Candida_glabrata_CBS138

Canis_familiaris

Cryptococcus_neoformans_var_JEC21

Debaryomyces_hansenii_CBS767

D_rerio

Drosophila_melanogaster

Encephalitozoon_cuniculi

Eremothecium_gossypii

Gallus_gallus

H_sepians

Kluyveromyces_lactis_NRRL_Y-1140

Macaca_mulatta

M_musculas

Pan_troglodytes

Plasmodium_falciparum

R_norvegicus

Saccharomyces_cerevisiae

Schizosaccharomyces_pombe

Strongylocentrotus_purpuratus

Tribolium_castaneum

Yarrowia_lipolytica_CLIB99

Bacteria

Acidobacteria_bacterium_Ellin345

Acidothermus_cellulolyticus_11B

Acinetobacter_sp_ADP1

Aeromonas_hydrophila_ATCC_7966

Aeropyrum_pernix

Agrobacterium_tumefaciens_C58_UWash

Alcanivorax_borkumensis_SK2

Alkalilimnicola_ehrlichei_MLHE-1

Anabaena_variabilis_ATCC_29413

Anaeromyxobacter_dehalogenans_2CP-C

Anaplasma_phagocytophilum_HZ

Aquifex_aeolicus

Archaeoglobus_fulgidus

Arthrobacter_FB24

Aster_yellows_witches-broom_phytoplasma_AYWB

Azoarcus_BH72

Bacillus_anthracis_str_Sterne

Bacteroides_fragilis_YCH46

Bartonella_henselae_Houston-1

Baumannia_cicadellinicola_Homalodisca_coagulata

Bdellovibrio_bacteriovorus

Bifidobacterium_longum

Bordetella_bronchiseptica

Borrelia_burgdorferi

Bradyrhizobium_japonicum

Brucella_melitensis

Buchnera_aphidicola_Sg

Burkholderia_xenovorans_LB400

Campylobacter_jejuni_RM1221

Candidatus_Pelagibacter_ubique_HTCC1062

Carboxydothermus_hydrogenoformans_Z-2901

Caulobacter_crescentus

Chlamydia_muridarum

Chlamydophila_felis_Fe_C-56

Chlorobium_phaeobacteroides_DSM_266

Chromobacterium_violaceum

Chromohalobacter_salexigens_DSM_3043

Clostridium_perfringens_ATCC_13124

Colwellia_psychrerythraea_34H

Corynebacterium_glutamicum_ATCC_13032_Kitasato

Coxiella_burnetii

Cyanobacteria_bacterium_Yellowstone_B-Prime

Cytophaga_hutchinsonii_ATCC_33406

Dechloromonas_aromatica_RCB

Dehalococcoides_CBDB1

Deinococcus_radiodurans

Desulfitobacterium_hafniense_Y51

Desulfotalea_psychrophila_LSv54

Desulfovibrio_desulfuricans_G20

Ehrlichia_chaffeensis_Arkansas

Enterococcus_faecalis_V583

Erwinia_carotovora_atroseptica_SCRI1043

Erythrobacter_litoralis_HTCC2594

Escherichia_coli_K12

Francisella_tularensis_novicida_U112

Frankia_alni_ACN14a

Fusobacterium_nucleatum

Geobacillus_kaustophilus_HTA426

Geobacter_metallireducens_GS-15

Gloeobacter_violaceus

Gluconobacter_oxydans_621H

Granulobacter_bethesdensis_CGDNIH1

Haemophilus_influenzae

Hahella_chejuensis_KCTC_2396

Haloarcula_marismortui_ATCC_43049

Halobacterium_sp

Haloquadratum_walsbyi

Helicobacter_hepaticus

Hyphomonas_neptunium_ATCC_15444

Idiomarina_loihiensis_L2TR

Jannaschia_CCS1

Lactobacillus_plantarum

Lactococcus_lactis

Lawsonia_intracellularis_PHE_MN1-00

Legionella_pneumophila_Philadelphia_1

Leifsonia_xyli_xyli_CTCB0

Leptospira_interrogans_serovar_Lai

Leuconostoc_mesenteroides_ATCC_8293

Listeria_monocytogenes_4b_F2365

Magnetococcus_MC-1

Magnetospirillum_magneticum_AMB-1

Mannheimia_succiniciproducens_MBEL55E

Maricaulis_maris_MCS10

Mesoplasma_florum_L1

Mesorhizobium_loti

Methanobacterium_thermoautotrophicum

Methanococcoides_burtonii_DSM_6242

Methanococcus_maripaludis_S2

Methanopyrus_kandleri

Methanosaeta_thermophila_PT

Methanosarcina_acetivorans

Methanosphaera_stadtmanae

Methanospirillum_hungatei_JF-1

Methylobacillus_flagellatus_KT

Methylococcus_capsulatus_Bath

Moorella_thermoacetica_ATCC_39073

Mycobacterium_smegmatis_MC2_155

Mycoplasma_penetrans

Myxococcus_xanthus_DK_1622

Nanoarchaeum_equitans

Natronomonas_pharaonis

Neisseria_meningitidis_Z2491

Neorickettsia_sennetsu_Miyayama

Nitrobacter_hamburgensis_X14

Nitrosococcus_oceani_ATCC_19707

Nitrosomonas_eutropha_C71

Nitrosospira_multiformis_ATCC_25196

Nocardia_farcinica_IFM10152

Nocardioides_JS614

Nostoc_sp

Novosphingobium_aromaticivorans_DSM_12444

Oceanobacillus_iheyensis

Oenococcus_oeni_PSU-1

Onion_yellows_phytoplasma

Parachlamydia_sp_UWE25

Paracoccus_denitrificans_PD1222

Pasteurella_multocida

Pediococcus_pentosaceus_ATCC_25745

Pelobacter_carbinolicus

Pelodictyon_luteolum_DSM_273

Photobacterium_profundum_SS9

Photorhabdus_luminescens

Picrophilus_torridus_DSM_9790

Pirellula_sp

Polaromonas_JS666

Porphyromonas_gingivalis_W83

Prochlorococcus_marinus_MIT9313

Propionibacterium_acnes_KPA171202

Pseudoalteromonas_atlantica_T6c

Pseudomonas_aeruginosa_UCBPP-PA14

Psychrobacter_cryohalolentis_K5

Psychromonas_ingrahamii_37

Pyrobaculum_aerophilum

Pyrococcus_abyssi

Ralstonia_eutropha_H16

Rhizobium_leguminosarum_bv_viciae_3841

Rhodobacter_sphaeroides_2_4_1

Rhodococcus_RHA1

Rhodoferax_ferrireducens_T118

Rhodopseudomonas_palustris_CGA009

Rhodospirillum_rubrum_ATCC_11170

Rickettsia_bellii_RML369-C

Roseobacter_denitrificans_OCh_114

Rubrobacter_xylanophilus_DSM_9941

Saccharophagus_degradans_2-40

Salinibacter_ruber_DSM_13855

Salmonella_typhimurium_LT2

Shewanella_oneidensis

Shigella_sonnei_Ss046

Silicibacter_pomeroyi_DSS-3

Sinorhizobium_meliloti

Sodalis_glossinidius_morsitans

Solibacter_usitatus_Ellin6076

Sphingopyxis_alaskensis_RB2256

Staphylococcus_aureus_Mu50

Streptococcus_agalactiae_A909

Streptomyces_avermitilis

Sulfolobus_solfataricus

Symbiobacterium_thermophilum_IAM14863

Synechococcus_elongatus_PCC_7942

Synechocystis_PCC6803

Syntrophobacter_fumaroxidans_MPOB

Syntrophomonas_wolfei_Goettingen

Syntrophus_aciditrophicus_SB

Thermoanaerobacter_tengcongensis

Thermobifida_fusca_YX

Thermococcus_kodakaraensis_KOD1

Thermofilum_pendens_Hrk_5

Thermoplasma_volcanium

Thermosynechococcus_elongatus

Thermotoga_maritima

Thermus_thermophilus_HB8

Thiobacillus_denitrificans_ATCC_25259

Thiomicrospira_crunogena_XCL-2

Treponema_denticola_ATCC_35405

Trichodesmium_erythraeum_IMS101

Tropheryma_whipplei_TW08_27

Ureaplasma_urealyticum

Vibrio_parahaemolyticus

Wigglesworthia_brevipalpis

Wolbachia_endosymbiont_of_Drosophila_melanogaster

Wolinella_succinogenes

Xanthomonas_campestris_vesicatoria_85-10

Xylella_fastidiosa_Temecula1

Yersinia_pseudotuberculosis_IP32953

Zymomonas_mobilis_ZM4
