# Supplementary figures and images for: Prediction of evolutionarily conserved interologs in Mus musculus
Source: BMC Genomics. 2008 Oct 8;9:465. doi: 10.1186/1471-2164-9-465 (PMC2571111; doi:10.1186/1471-2164-9-465)

## Slide 1
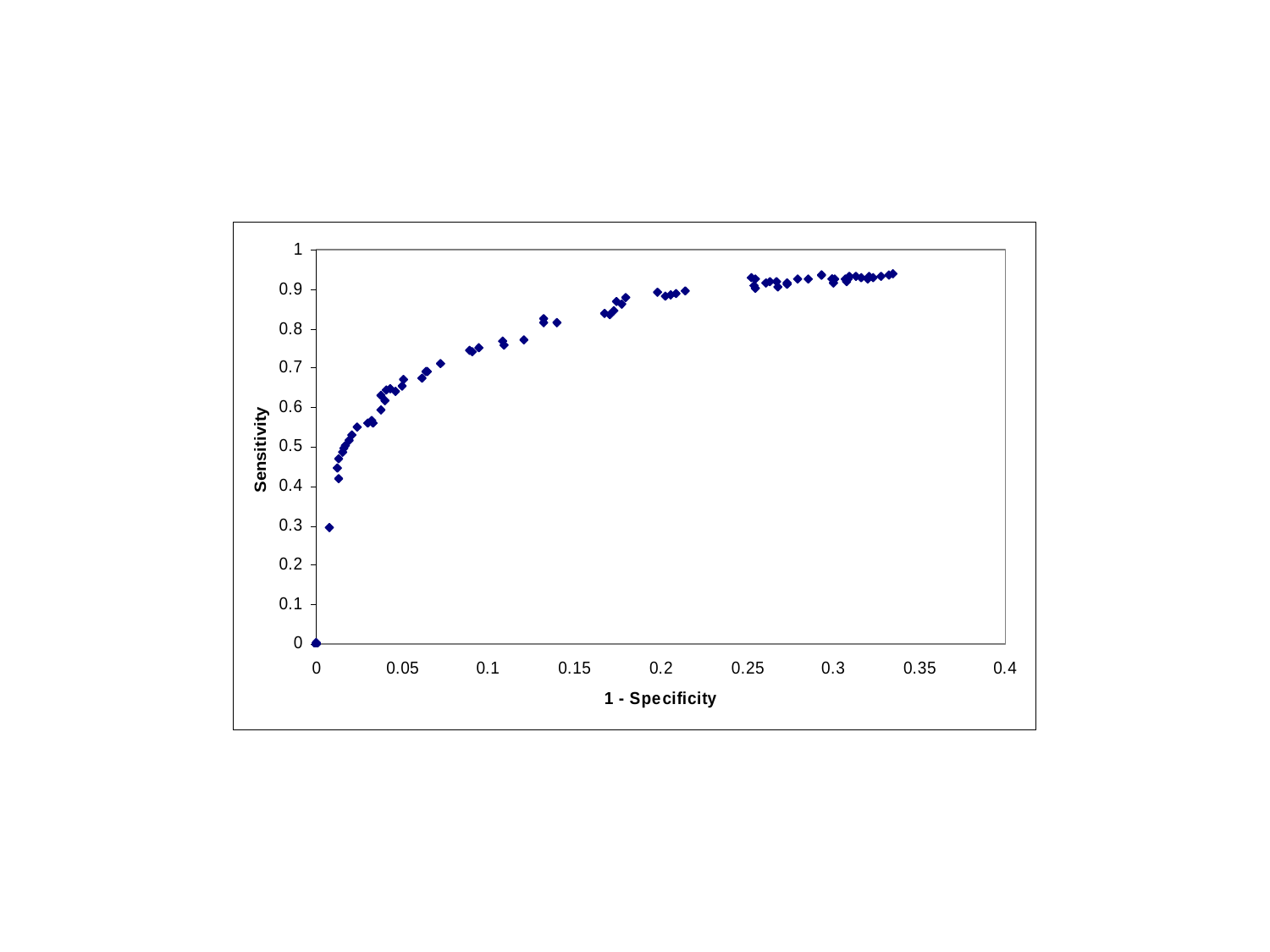

Supplement: Additional file 4 — ROC Graph for prediction of protein – protein interactions. The ROC curve is a plot of "sensitivity (True positive rate)" versus "1 – specificity (False positive rate)." The "sensitivity" and "1 – specificity" values at the point near the upper left corner are 0.82 and 0.13, respectively, where the balanced accuracy for prediction of protein – protein interaction reaches the maximum (86%). [file 1471-2164-9-465-S4.ppt]
