# Supplementary material for: Prediction of evolutionarily conserved interologs in Mus musculus
Source: BMC Genomics. 2008 Oct 8;9:465. doi: 10.1186/1471-2164-9-465 (PMC2571111; doi:10.1186/1471-2164-9-465)
Supplement: Additional file 5 — Accuracy of cross validation in phylogenetic profiles. A table shows the number of true positives (TP), false negatives (FN), true negatives (TN), and false positives (FP) in each step of five-fold cross validation trials. Sensitivity and Specificity are calculated according to the formula described in the Methods section. The accuracy reaches the maximum in the 4th step of cross validation trials. [file 1471-2164-9-465-S5.doc]

**Supplemental Table 6. Cross validation accuracy of phylogenetic profiles**

|  | TP | FN | TN | FP | Sensitivity | Specificity | Accuracy |
| --- | --- | --- | --- | --- | --- | --- | --- |
| 1^st^ Trial | 909 | 212 | 779 | 111 | 0.81 | 0.87 | 84.30 |
| 2^nd^ Trial | 902 | 219 | 768 | 122 | 0.80 | 0.86 | 83.37 |
| 3^rd^ Trial | 905 | 216 | 775 | 115 | 0.80 | 0.87 | 83.90 |
| 4^th^ Trial | 926 | 195 | 774 | 116 | 0.82 | 0.86 | 84.78 |
| 5^th^ Trial | 927 | 194 | 765 | 125 | 0.82 | 0.85 | 84.32 |
| Average | 913.8 | 207.2 | 772.2 | 117.8 | 0.81 | 0.86 | 84.14 |

Legend: TP, true positives; FN, false negatives; TN, true negatives; FP, false positives. Sensitivity and Specificity are calculated according to the formula described in the Methods section.
